# Aqueous humor cytokines levels and illuminated microcatheter-assisted circumferential trabeculotomy outcome in open-angle glaucoma patients: a 24-month prospective study

**DOI:** 10.3389/fimmu.2025.1575909

**Published:** 2025-05-20

**Authors:** Tingyi Wu, Qianqian Ji, Yanfeng Liao, Xiaoyu Zhao, Ying Hong

**Affiliations:** ^1^ Department of Ophthalmology, Peking University Third Hospital, Beijing, China; ^2^ Beijing Key Laboratory of Restoration of Damaged Ocular Nerve, Peking University Third Hospital, Beijing, China; ^3^ Tianjin Key Laboratory of Retinal Functions and Diseases, Tianjin Branch of National Clinical Research Center for Ocular Disease, Eye Institute and School of Optometry, Tianjin Medical University Eye Hospital, Tianjin, China

**Keywords:** illuminated microcatheter-assisted circumferential trabeculotomy, open-angle glaucoma, aqueous humor, cytokines, inflammation

## Abstract

**Purpose:**

To investigate the influence of aqueous humor cytokines levels on the failure of illuminated microcatheter-assisted circumferential trabeculotomy (MAT) in open-angle glaucoma (OAG) patients.

**Methods:**

This was a prospective case series with a follow-up period of 24 months. General information and ocular examinations were recorded. Aqueous humor was collected at the time of surgery. Eight aqueous humor cytokines were analyzed: CCL2, VCAM-1, ICAM-1, IL-6, IL-8, IL-10, CXCL10 and G-CSF. Bioinformatics analysis was used to explore the protein network and the possible pathways related to OAG. Surgical failure was defined as a requirement for glaucoma reoperation or intraocular pressure (IOP) greater than 21mmHg with more than 3 topical antiglaucoma medications at 24-month follow-up.

**Results:**

Sixty-five eyes were enrolled (58 success and 7 failure). The levels of CCL2, ICAM-1, IL-6 and CXCL10 in aqueous humor were significantly higher in the surgical failure group (*P* = 0.024, 0.002, 0.022 and 0.008, respectively). A higher percentage of secondary glaucoma (*P* < 0.001), younger age (*P* = 0.019), worse preoperative BCVA (*P* = 0.022), higher preoperative IOP (*P* = 0.022) and more preoperative topical antiglaucoma medications (*P* = 0.029) were significantly observed in the surgical failure group. Bioinformatics analysis identified 4 hub proteins, including CCL2, CXCL10, IL-6 and CXCR3, and demonstrated the potential role of chemokine signaling pathway in MAT surgical outcome.

**Conclusion:**

Higher concentrations of CCL2, ICAM-1, IL-6 and CXCL10 in the aqueous humor were related to the failure of MAT surgery in OAG patients, and chemokine signaling pathway might be associated with the surgical outcome.

## Introduction

Glaucoma is a group of chronic progressive optic neuropathy characterized by degeneration of retinal ganglion cells and damage to optic nerve (1, 2). Worldwide, around 68.4% of cases are open-angle glaucoma (OAG), making it the most common category (3). Surgical interventions are often necessary to achieve better control of intraocular pressure (IOP) in advanced OAG cases (4). Minimally invasive glaucoma surgeries (MIGS) have become widely used due to the faster recovery and fewer complications compared with traditional glaucoma surgeries (5, 6). The illuminated microcatheter-assisted circumferential trabeculotomy (MAT) has been applied in recent years in the treatment of glaucoma. MAT not only possesses the advantages of MIGS but also provides direct visualization of Schlemm’s canal, which enables surgeons to perform circumferential trabeculotomy more safely and completely (7).

Previous studies demonstrated that intraocular cytokines were related to the outcome of glaucoma surgeries. Jung et al. proved that the levels of aqueous humor cytokines were associated with the success rate of Ahmed glaucoma valve implantation (8), and Burgos-Blasco et al. found that the cytokines levels in aqueous humor could serve as a prognostic factor for OAG patients receiving trabeculectomy (9). Therefore, we infer that the level of intraocular inflammatory condition may also influence MAT surgical failure rate. To the best of our knowledge, there is still a lack of research analyzing the relationship between aqueous humor cytokines levels and surgical outcome in OAG patients receiving MAT. We aim to compare the difference in aqueous humor cytokines levels between the surgical success group and failure group in order to explore the influence of aqueous humor inflammatory condition on MAT outcome.

## Materials and methods

### Study design

The present study was a prospective case series. All patients who visited Peking University Third Hospital Ophthalmology Department from March 2022 to August 2023 were included when they met the following criteria which were formulated according to previous studies (8, 10, 11). The inclusion criteria included: (1) diagnosed with OAG (including primary glaucoma, congenital glaucoma and secondary glaucoma) using gonioscopy and ultrasound biomicroscopy (UBM), (2) preoperative IOP greater than 15mmHg despite receiving maximum antiglaucoma medications, (3) underwent MAT surgery without postoperative complications. The exclusion criteria included: (1) combined with severe systemic diseases, (2) pregnant or under breastfeeding, (3) unable to consent and follow-up. All patients were followed up for at least 24 months. The research followed the tenets of the Declaration of Helsinki and was approved by the Ethics Committee of Peking University Third Hospital (Approved No. IRB 201416605). Written informed consent was obtained from each patient.

### Preoperative and postoperative evaluation

Patients’ age, sex, medical history and antiglaucoma medications were recorded. Ophthalmic examinations included best corrected visual acuity (BCVA, logMAR), Goldmann applanation tonometry (GAT), slit lamp biomicroscopy, gonioscopy, fundus examination and UBM (SUOER SW-3200L, Tianjin, China).

Postoperative examinations were performed at 1 day, 1 week, 1 month, 6 months and 24 months after surgery. BCVA, GAT, slit lamp biomicroscopy and fundus examination were performed at each follow-up.

### Surgical procedures

All surgeries were accomplished by a single experienced surgeon (Y. H) and were performed under general anesthesia. Approximately 100 μL of aqueous humor was collected before surgery. A fornix-based conjunctival flap was made. A superficial scleral flap with an approximate size of 5 × 4 × 3-mm was made under the conjunctival flap. Then, a 2 × 2-mm deep scleral flap was made. The external wall of the Schlemm’s canal was incised and its ostia was exposed. Afterwards, the Schlemm’s canal was cannulated by introducing a microcatheter (iTrack™, Nova Eye Medical Limited, Kent Town, Australia) through the ostia, and the cannulated degree was recorded. Trabeculotomy was performed by pulling the two ends of the catheter. 10/0 nylon sutures were used to close the scleral and conjunctival flaps. For patients who needed a combination of cataract surgery, standard phacoemulsification with intracapsular IOL implantation (CENTURION^®^ Vision System, Alcon, Texas, U.S.) was performed after the superficial scleral flaps were made.

### Aqueous humor cytokine analysis

All aqueous humor samples were sealed in Eppendorf tubes and preserved at −80°C freezers. The aqueous humor cytokines were analyzed using the BD Cytometric Bead Array Flex Set System kits (BD Biosciences, New Jersey, U.S.) in conjunction with the DxFLEX flow cytometer (Beckman Coulter, Indiana, U.S.) according to the manufacturer’s instructions. The levels of the following cytokines were determined: chemokine (CC-motif) ligand 2 (CCL2); C-X-C motif chemokine 10 (CXCL10); granulocyte-colony stimulating factor (G-CSF); intercellular adhesion molecule-1 (ICAM-1); interleukin-6 (IL-6); interleukin-8 (IL-8); interleukin-10 (IL-10); vascular cell adhesion molecule-1 (VCAM-1). The assay sensitivity and limits of detection for each cytokine were as follows (according to the manufacturer’s instructions): CCL2: 1.3 pg/mL, CXCL10: 0.5 pg/mL, G-CSF: 1.6 pg/mL, ICAM-1: 25.7 pg/mL, IL-6: 1.6 pg/mL, IL-8: 1.2 pg/mL, IL-10: 0.13 pg/mL and VCAM-1: 12.2 pg/mL.

### Outcome measurement

The postoperative patients were divided into surgical success group and failure group. Surgical failure was defined as a requirement for glaucoma reoperation or IOP greater than 21mmHg with more than 3 supplemental topical antiglaucoma medications at 24-month follow-up (8). Reoperation was defined as a second glaucoma surgery, such as trabeculectomy, glaucoma drainage implant surgeries and cyclodestructive procedures. The surgical success rate and failure rate were calculated and recorded.

### Bioinformatics analysis

To explore the protein network and mechanisms, we performed protein-protein interaction (PPI) analysis using STRING 12.0 database (http://string-db.org). The minimum required interaction score was set as medium confidence (0.400) and the max number of interactors was 50. The active interaction sources included textmining, experiments, databases, neighborhood, gene fusion, co-expression and co-occurrence. The degree of each node was further calculated using NetworkAnalyzer in Cytoscape (https://cytoscape.org, version 3.9.1). Furthermore, we used cytoHubba in Cytoscape to explore hub proteins in the PPI network. Four topological algorithms, including Maximal Clique Centrality (MCC), Maximum Neighborhood Component (MNC), Edge Percolated Component (EPC) and Degree, were applied separately to calculate and select the top 10 important nodes in the PPI network. The results were displayed in the Venn diagram (https://www.vandepeerlab.org) to execute Overlap analysis. To explore the potential metabolic and signaling pathways, we further performed Gene Ontology (GO) enrichment analysis and Kyoto Encyclopedia of Genes and Genomes (KEGG) pathway analysis (http://string-db.org). GO enrichment analysis was performed based on three categories, including biological process (BP), molecular function (MF) and cellular component (CC).

### Statistical analysis

Statistical analysis was performed using SPSS (version 29.0, IBM Corp., New York, U.S.). Independent variables were represented as mean ± standard deviation (SD) or quartiles according to the normality of the data. Differences in categorical data among the 2 groups were analyzed using the chi-square test. The comparison of continuous variables among the two groups was accomplished by the student *t* test or Mann-Whitney *U* test according to the normality of the data. *P* value of less than 0.05 was considered statistically significant differences.

## Results

A total of 65 eyes of 65 patients (58 eyes success and 7 eyes failure) were enrolled. Preoperative demographic characteristics are summarized in **Table 1**. All eyes received MAT surgery, and 19 of them were combined with cataract surgery.

**Table 1:** Demographic characteristics.

Characteristic	Mean ± SD	Range (min, max)
Age (years)	49.4 ± 19.3	1, 77
Sex (male/female)	45/20	
BCVA (logMAR)	1.0 ± 0.9	0, 2.4
IOP (mmHg)	27.4 ± 8.1	17, 50
Glaucoma type		
POAG	41 (63%)	
PCG	4 (6%)	
Secondary OAG	17 (26%)	
PSS	3 (5%)	
Amount of topical antiglaucoma medications	2.9 ± 1.0	1, 4
1	4 (6%)	
2	22 (34%)	
3	13 (20%)	
4	26 (40%)	

BCVA, best corrected visual acuity; IOP, intraocular pressure; OAG, open-angle glaucoma; PCG, primary congenital glaucoma; POAG, primary open-angle glaucoma; PSS, Posner-Schlossman syndrome; SD, standard deviation.

The comparisons of clinical features between surgical success and failure groups are summarized in **Table 2**. The results showed that the age was significantly smaller in the surgical failure group (*P* = 0.019). A statistically higher percentage of primary glaucoma was present in the surgical success group than the failure group (*P* < 0.001). Significantly better BCVA (*P* = 0.022), lower IOP (*P* = 0.022) and fewer topical glaucoma medications (*P* = 0.029) were observed in the success group than failure group preoperatively. Other clinical parameters did not demonstrate statistical differences between the groups.

**Table 2:** Clinical features in surgical success and failure groups.

Characteristic	Success	Failure	*χ²/Z*	*P*
Age (years)	51.7 ± 17.8	30.6 ± 22.8	-2.339	0.019*
Sex (male/female)	40/18	5/2	0.018	0.894
Glaucoma type (primary/secondary)	48/10	0/7	22.150	< 0.001*
Combination with cataract surgery (yes/no)	19/39	0/7	3.240	0.072
Range of incision (degree)	317.24 ± 90.14	235.71 ± 155.33	-1.526	0.127
Preoperative BCVA (logMAR)	0.9 ± 0.9	1.7 ± 0.8	-2.289	0.022*
Preoperative IOP (mmHg)	26.6 ± 7.7	33.9 ± 8.7	-2.291	0.022*
Amount of preoperative topical antiglaucoma medications	2.8 ± 1.0	3.7 ± 0.5	-2.188	0.029*

BCVA, best corrected visual acuity; IOP, intraocular pressure.

*P < 0.05.

The boldface indicates statistical significance.

The comparisons of aqueous humor cytokines concentrations between surgical success and failure groups are summarized in **Table 3**. Among the aqueous humor cytokines, the concentration of CCL2, ICAM-1, IL-6 and CXCL10 were statistically lower in the surgical success group than the failure group (*P* = 0.024, 0.002, 0.022 and 0.008, respectively). Other aqueous humor cytokines did not show significant differences between the groups.

**Table 3:** Aqueous humor cytokines levels in surgical success and failure groups.

Cytokine	Success	Failure	*Z*	*P*
CCL2 (pg/mL)	413.53 (309.91, 869.81)	971.22 (662.73, 1804.21)	-2.257	0.024*
VCAM-1 (pg/mL)	4248.83 (1215.91, 10961.24)	11237.35 (6051.26, 58231.47)	-1.843	0.065
ICAM-1 (pg/mL)	349.53 (166.54, 912.05)	3020.89 (2415.37, 25491.15)	-3.109	0.002*
IL-6 (pg/mL)	6.58 (2.00, 377.17)	294.14 (76.98, 1976.00)	-2.286	0.022*
IL-8 (pg/mL)	22.83 (6.76, 102.72)	37.01 (18.43, 104.42)	-0.772	0.440
IL-10 (pg/mL)	0.00 (0.00, 0.00)	0.00 (0.00, 0.50)	-1.717	0.086
CXCL10 (pg/mL)	76.94 (22.64, 220.29)	317.79 (203.15, 549.50)	-2.660	0.008*
G-CSF (pg/mL)	0.00 (0.00, 2.40)	2.21 (0.00, 13.47)	-1.311	0.190

CCL2, chemokine (CC-motif) ligand 2; CXCL10, C-X-C motif chemokine 10; G-CSF, granulocyte-colony stimulating factor; ICAM-1, intercellular adhesion molecule-1; IL, interleukin; VCAM-1, vascular cell adhesion molecule-1.

The results in **Table 3** were presented in quartile, i.e., median (25th percentile, 75th percentile).

*P < 0.05.

The boldface indicates statistical significance.

**Figure 1:**
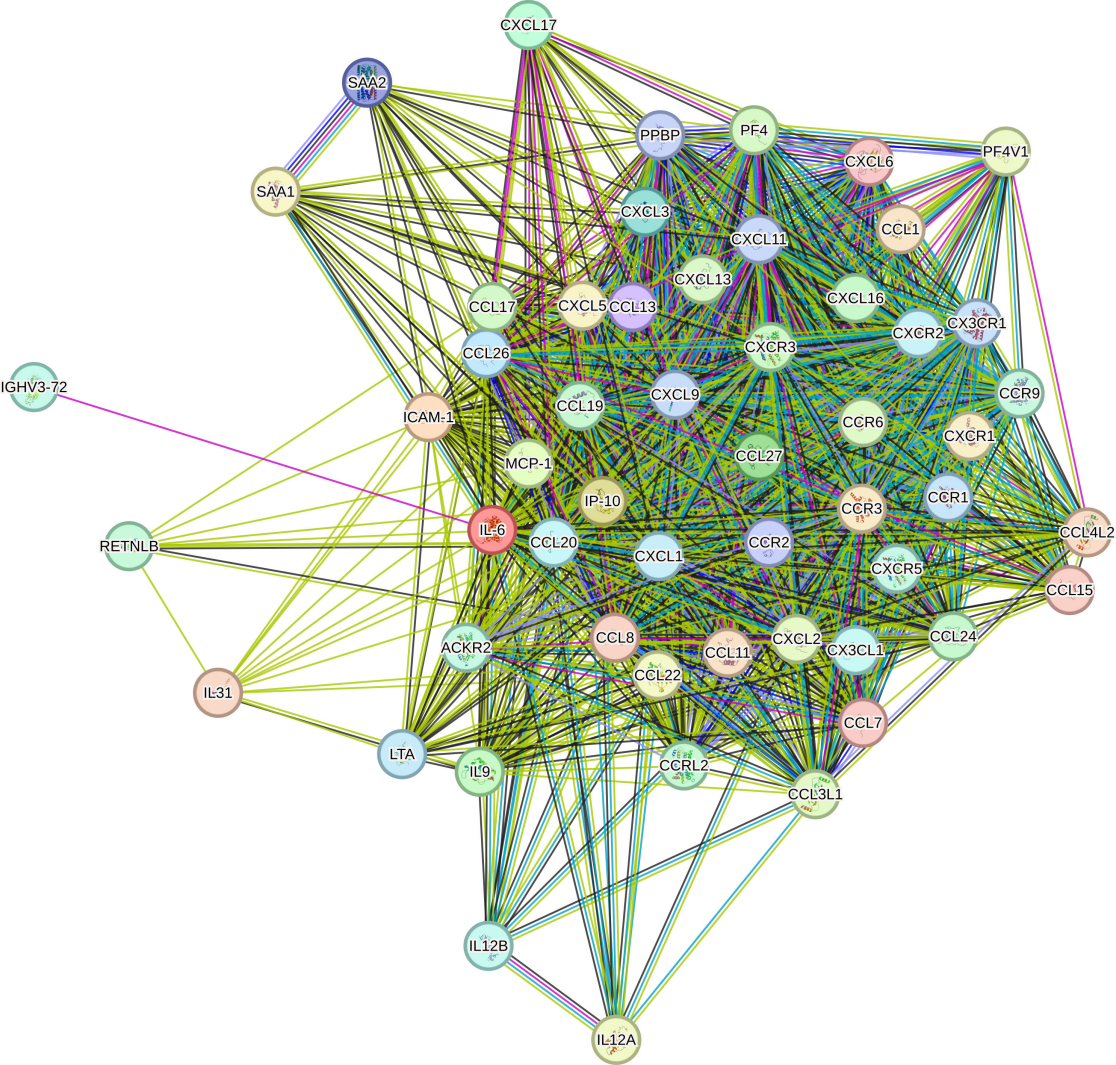
The protein-protein interaction network. The nodes represent different proteins. The edges represent interactions exist between two proteins. The light blue edges represent known interactions from curated databases and the pink edges represent known interactions determined experimentally. Predicted interactions include green edges representing gene neighborhood, red edges representing gene fusions and blue edges representing gene co-occurrence. Other interactions include light green edges representing textmining, black edges representing co-expression and purple edges representing protein homology.

**Figure 2:**
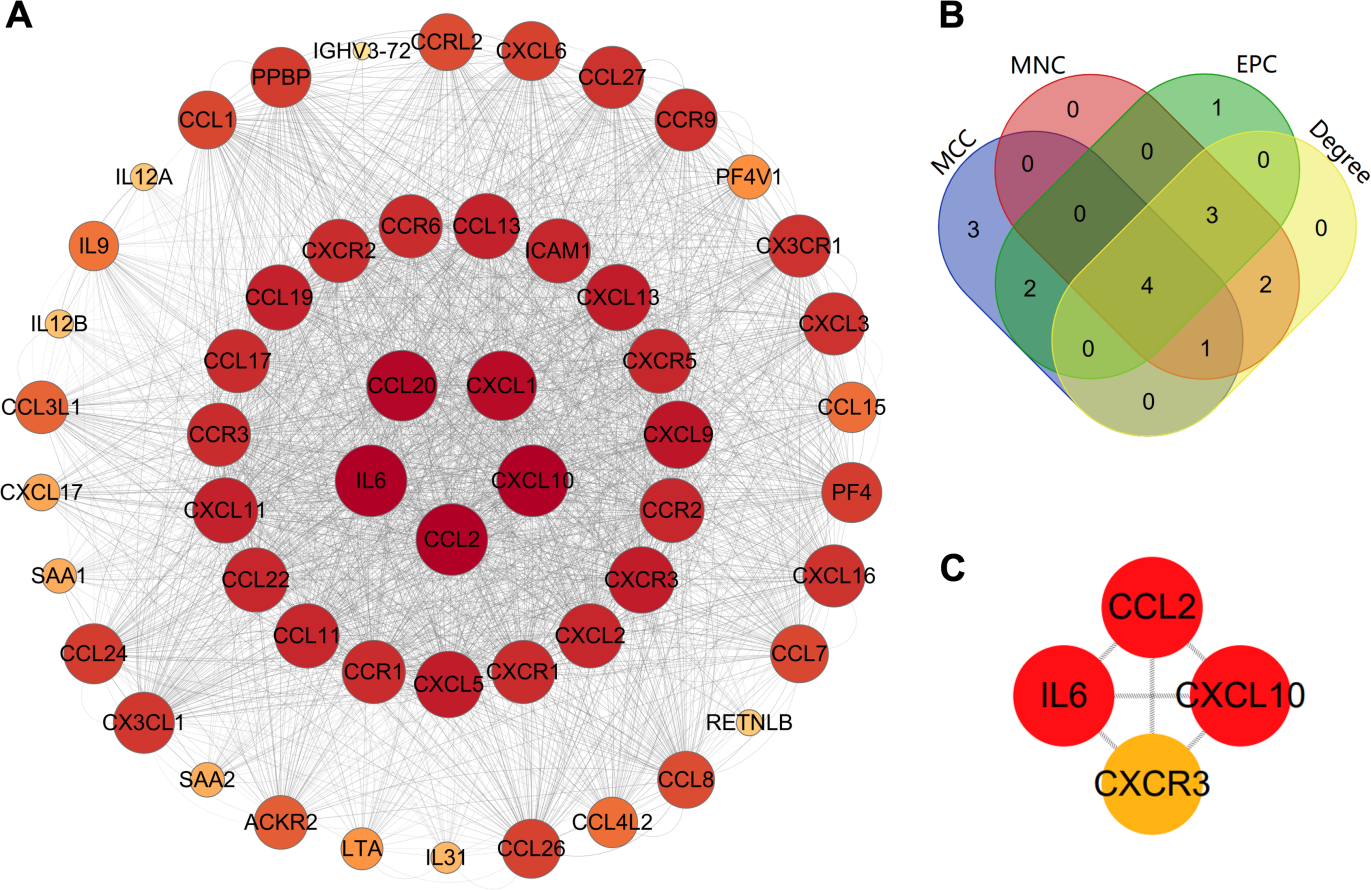
Identification of hub proteins. **(A)** The protein-protein interaction network reconstructed by Cytoscape; **(B)** The Venn diagram of the top 10 important nodes calculated by MCC, MNC, EPC and Degree algorithms in cytoHubba; **(C)** The hub proteins. EPC, Edge Percolated Component; MCC, Maximal Clique Centrality; MNC, Maximum Neighborhood Component.

**Figure 3:**
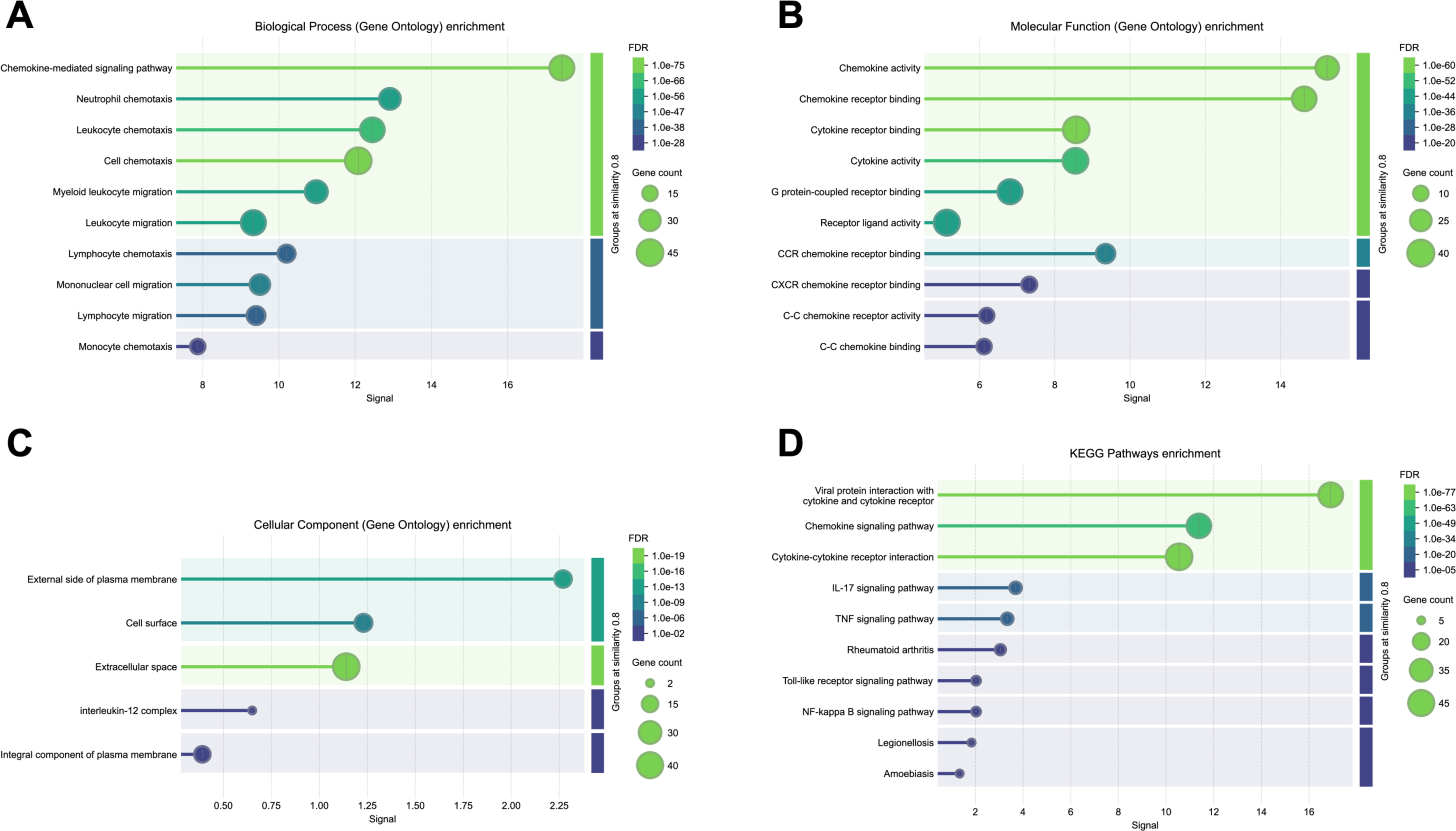
Gene ontology (GO) enrichment analysis and Kyoto Encyclopedia of Genes and Genomes (KEGG) pathway analysis. The color scale represents the false discovery rate (FDR) describing the significant level of enrichment. The dots represent gene count describing the number of proteins in the network annotated with a particular term. The horizontal axis represents the signal defined as a weighted harmonic mean between the observed/expected ratio and -log (FDR). The terms are grouped by similarity ≥ 0.8 and sorted by signal. **(A)** Biological Process (BP, GO) enrichment; **(B)** Molecular Function (MF, GO) enrichment; **(C)** Cellular Component (CC, GO) enrichment; **(D)** KEGG pathways enrichment.

The PPI network is demonstrated in **Figure 1**. The results showed that there were 50 proteins interacting with CCL2, ICAM-1, IL-6 and CXCL10. These formed a network with 54 nodes and 1022 edges. The results of Cytoscape NetworkAnalyzer demonstrated that the median degree of all nodes was 86. CCL2, CXCL10 and IL-6 had the largest node degree of 104, and the degree of ICAM-1 was 90. The results of cytoHubba and Venn diagram analysis are demonstrated in **Figure 2**. Four hub proteins, including CCL2, CXCL10, IL-6 and CXCR3, were obtained. The results of functional enrichment are shown in **Figure 3**. In GO functional annotation and enrichment analysis, 227 terms in BP, 29 terms in MF and 5 terms in CC were significantly enriched. In KEGG pathway analysis, 27 pathways were significantly enriched. The primary terms in GO analysis were related to chemokine signaling pathway, inflammatory cells chemotaxis and migration. The primary pathways in KEGG enrichment were viral protein interaction with cytokine and cytokine receptor, chemokine signaling pathway and cytokine-cytokine receptor interaction.

## Discussion

To the best of our knowledge, this is the first study to simultaneously analyze these 8 cytokines in Chinese OAG eyes with MAT surgery. The present study demonstrated that higher concentrations of CCL2, ICAM-1, IL-6 and CXCL10 in the aqueous humor were associated with a greater likelihood of MAT surgical failure. Other demographic and clinical features correlated with surgical failure included younger age, secondary glaucoma, worse visual acuity, higher preoperative IOP and greater amount of preoperative topical glaucoma medications.

The surgical failure group had a significantly higher proportion of secondary OAG, which was consistent with the study of Aktas et al. reporting that the IOP reduction in secondary OAG patients receiving trabeculotomy was worse than primary ones at the 18-month follow-up (12). Other possible reasons for a higher surgical failure rate for secondary OAG patients were the active inflammation caused by underlying diseases and more antiglaucoma eyedrops used preoperatively (12, 13). Other risk factors, including younger age (14), worse preoperative BCVA (15), higher preoperative IOP (16) and more preoperative topical antiglaucoma medications (16) were also in accordance with previous studies.

It has been suggested that the aqueous humor of glaucomatous eyes had greater concentrations of several growth factors and inflammatory cytokines (8, 17), and these cytokines might be responsible for poor outcome after glaucoma surgeries (18). Jung et al. investigated the levels of cytokines in the aqueous humor collected at the time of Ahmed Glaucoma Valve implantation surgeries and found that higher levels of TGF-ß2 and CCL2 were associated with surgical failure (8). It was also observed that increases in some growth factors and cytokines in the aqueous humor would enhance the wound healing response after traditional filtration surgery, i.e., trabeculectomy (19). These cytokines played key roles in inflammation leading to severe fibrogenesis and proliferation after filtration surgeries. In recent years, the proportion of internal drainage surgeries increased rapidly due to the safety, minimally invasive and avoidance of bleb-related postoperative complications compared with traditional incisional surgeries (6). As a kind of MIGS surgery, the widespread use of internal drainage surgeries has made it necessary to study their relationship with inflammatory cytokines.

Higher CCL2 level in the aqueous humor were correlated with MAT surgical failure. The CCL2 belongs to the C-C chemokine family and is one of the key chemokines that regulate the migration of monocytes/macrophages (20). It has been found that CCL2 was involved in the recruitment of blood-derived monocytes into the sites of active inflammation (21). Previous research has suggested that CCL2 might play an important role in several inflammatory diseases, e.g., inflammatory bowel disease, rheumatoid arthritis and asthma (20). Similarly, Freedman et al. demonstrated that OAG patients had a greater level of CCL2 in their aqueous humor indicating the potential relationship between OAG and inflammation (22). Therefore, it may be inferred that the chronic inflammation activated by a greater level of CCL2 could cause reobstruction of trabecular meshwork despite being incised successfully by MAT surgery and result in a higher possibility of surgical failure.

In our study, we observed that the surgical failure group had a statistically greater concentration of IL-6 compared with the success group. IL-6 is a pro-inflammatory cytokine, which is closely related to chronic inflammation and immunomodulation (23). The influence of IL-6 on glaucoma remains controversial. Freedman et al. demonstrated that a higher level of IL-6 in the aqueous humor was observed in OAG patients (22). It was suggested that IL-6 may play a role in regulating the outflow facility through the trabecular meshwork. The mechanical stress produced by elevated IOP induced the expression of IL-6 aiming to maintain the homeostasis of IOP value (24, 25). On the other hand, IL-6 would affect the function of the blood-aqueous barrier in OAG patients, resulting in the leakage of various inflammatory cells into the aqueous humor (9, 26). Elevated level of anterior segment inflammation would have a potential negative effect on surgical success rate.

Surgical failure had relevance to an elevated ICAM-1 level in aqueous humor. ICAM-1 is a crucial mediator in the inflammatory process. It is up-regulated in the vascular endothelial cells when there is active inflammation and is responsible for regulating the migration of leukocytes crossing the vessel wall (27). ICAM-1 was identified to be produced by trabecular meshwork cells under oxidative stress which was a mechanism of glaucoma. Additionally, ICAM-1 was the major protein in several signaling pathways related to the apoptosis of trabecular meshwork cells (28). Therefore, elevated concentration of ICAM-1 might be associated with anterior segment inflammation and oxidative stress inducing a higher possibility of surgical failure.

A statistically higher level of CXCL10 was also identified in current research. CXCL10 is a chemokine that plays a role in promoting chemoattraction for inflammatory cells (29). Previous research has shown that IOP was positively associated with the level of CXCL10 in aqueous humor in POAG patients (30). In animal research investigating the mouse model of acute glaucoma, Ha et al. found that the CXCL10/CXCR3 pathway, which is involved in inflammatory cell recruitment and activation, was upregulated (31). The result demonstrated the potential effect of excessive inflammation in inducing tissue injury, and this might result in an increased surgical failure rate.

The PPI network demonstrated the complexity of interaction between different cytokines. Four hub proteins, including CCL2, CXCL10, IL-6 and CXCR3, were identified after calculation by Cytoscape, and three of them were chemokines. It is noteworthy that the hub proteins are not entirely identical to our study results. The hub proteins obtained from bioinformatics analysis refer to proteins that have the greatest interactions and occupy hub positions in the PPI network. They are probably not the most differentially expressed proteins which reflects changes in protein levels under a specific condition. Hub proteins are defined based on their centrality and regulatory roles within the network, and play crucial roles in signal transduction and regulation through extensive interactions with other proteins. They potentially serve as regulatory nodes in pathological processes rather than merely represent the final effects. In enrichment analysis, both GO and KEGG methods identified chemokine signaling pathway. Wang et al. observed significant changes of chemokine signaling pathway in retinal ganglion cells response to elevated IOP (32). In another study, Denoyer et al. found that CXCR3 antagonist could restore trabecular function and protect retinal neurons (33). Therefore, it could be inferred that the upregulation of some chemokines could have negative effects on glaucoma. However, the specific effects of hub proteins and different pathways on OAG and surgical outcome still require further basic research.

There are some limitations in our study. First, the levels of aqueous humor cytokines are affected by other factors, e.g., glaucoma type (12, 13, 34), preoperative antiglaucoma medications used (35) and the prior therapeutic procedures (35). Further studies are required to investigate these factors on MAT outcome. Second, due to the limited volume of aqueous humor available, a number of aqueous humor cytokines were not assessed, and these may also influence the surgical outcome. The cytokines we selected for measurement were based on previous studies that suggested their potential association with inflammatory processes in glaucomatous eyes. As a result, the measured cytokines may not fully overlap with the hub proteins identified in the bioinformatics analysis. For example, CXCR3 was identified as a hub protein through bioinformatics analysis, but it was not directly measured in our study. Given the established interaction between CXCR3 and CXCL10 (31), which was significantly elevated in surgical failures, it is possible that the CXCL10/CXCR3 signaling axis plays a role in MAT outcomes. Future studies are warranted to directly investigate the role of CXCR3 and other potentially relevant cytokines in aqueous humor. Third, the results of our study primarily reveal the differences in aqueous humor cytokines levels between the two groups, and their exact influence on surgical outcome need further investigation. Additionally, the results of bioinformatics analysis primarily reflect general pathways, which may not fully reflect the condition in the aqueous humor since the anterior chamber is immune-privileged. These also warrant investigation in the future.

In conclusion, significantly higher levels of CCL2, ICAM-1, IL-6 and CXCL10 in the preoperative aqueous humor were found in MAT surgical failure patients, which implied the potential influence of inflammation on surgical outcome. Further studies may be warranted to investigate whether monoclonal antibodies targeting these cytokines could improve the success rate of MAT surgery in OAG patients.

## Data Availability

The raw data supporting the conclusions of this article will be made available by the authors, without undue reservation.
